# Constituents of French Marigold (*Tagetes patula* L.) Flowers Protect Jurkat T-Cells against Oxidative Stress

**DOI:** 10.1155/2016/4216285

**Published:** 2016-06-28

**Authors:** Irakli Chkhikvishvili, Tamar Sanikidze, Nunu Gogia, Maia Enukidze, Marine Machavariani, Nana Kipiani, Yakov Vinokur, Victor Rodov

**Affiliations:** ^1^Institute of Medical Biotechnology, Tbilisi State Medical University, 33 Vazha Pshavela Avenue, 0177 Tbilisi, Georgia; ^2^Department of Postharvest Science of Fresh Produce, Agricultural Research Organization, The Volcani Center, HaMaccabim Road 68, P.O. Box 15159, 7528809 Rishon LeZion, Israel

## Abstract

The flowers of French marigold (*Tagetes patula* L.) are widely used in folk medicine, in particular for treating inflammation-related disorders. However, cellular mechanisms of this activity demand further investigation. In the present work, we studied the potential of* T. patula* compounds to alleviate the oxidative stress in hydrogen peroxide-challenged human lymphoblastoid Jurkat T-cells. Crude extracts of marigold flowers and purified fractions containing flavonoids patuletin, quercetagetin, and quercetin and their derivatives, as well as the carotenoid lutein, were brought in contact with Jurkat cells challenged with 25 or 50 *μ*M H_2_O_2_. Hydrogen peroxide caused oxidative stress in the cells, manifested as generation of superoxide and peroxyl radicals, reduced viability, arrested cell cycle, and enhanced apoptosis. The stress was alleviated by marigold ingredients that demonstrated high radical-scavenging capacity and enhanced the activity of antioxidant enzymes involved in neutralization of reactive oxygen species. Flavonoid fraction rich in quercetin and quercetagetin showed the highest cytoprotective activity, while patuletin in high dose exerted a cytotoxic effect associated with its anticancer potential.* T. patula* compounds enhanced the production of anti-inflammatory and antioxidant interleukin-10 (IL-10) in Jurkat cells. Both direct radical-scavenging capacity and stimulation of protective cellular mechanisms can underlay the anti-inflammatory properties of marigold flowers.

## 1. Introduction

The genus* Tagetes* (Asteraceae) is native to Americas but some of its members (in particular* T. erecta* and* T. patula*) commonly known as marigolds were naturalized in the Old World (India, North Africa, and Europe) as early as in 16th century [1]. Moreover, some researchers suggest that both species reached India anciently through pre-Columbian transoceanic voyages [2]. Marigold was introduced to Georgia from India, and its ground dried petals became one of the most popular local spices [3]. Both* T. erecta* and* T. patula* are grown in Georgia as spice and dye plants [4] recognized for their health-beneficial properties [5].

*Tagetes* is a multipurpose plant having ornamental, ritual, medicinal, anthelmintic, insecticidal, colorant, food, and forage applications [6, 7]. Healing properties of* Tagetes* species have been implemented by folk medicine for centuries [8]. In particular, flowers and entire herb of* T. patula* (French marigold) are used for preparing ethnobotanical remedies against rheumatism, stomach and intestinal problems, kidney and hepatic disorders, fever, and pneumonia [6, 9]. The infusion of* T. patula* flowers is also implemented as eyewash [6]. The efficacy of orally administered methanolic extracts of* T. patula* florets against acute and chronic inflammation was confirmed in experiments with animal models [10]. Similar results were obtained for* T. erecta* (African marigold) extracts [11]. Furthermore, a double-blind placebo-controlled clinical trial showed effectiveness of marigold therapy using* T. patula* preparations in treating human inflammation-associated disorders such as bunion [12]. The anti-inflammatory effect of* T. patula* extracts could be reproduced in animal model by oral administration of its flavonoid constituents, patuletin and patulitrin [13]. Lipophilic ingredients of marigold flowers, the carotenoid lutein and essential oil compounds, were also reported to possess anti-inflammatory properties [14, 15]. In our previous study, both hydrophilic and lipophilic fractions from* T. patula* petals showed the highest radical-scavenging capacities among all Georgian spices tested [16].

However, the cellular mechanisms by which the marigold extracts exert their anti-inflammatory effects are not fully understood and demand further investigation. Methanol extracts of* T. patula* flowers as well as isolated patuletin were reported to scavenge peroxyl and superoxide radicals in chemical systems and in human neutrophils and at the same time to exert cytotoxic and growth inhibitory effects towards a range of human cancer cell lines, in particular HeLa cells [17]. On the other hand, ethanolic and ethyl acetate extracts of marigold flowers showed no cytotoxicity towards H460 lung cancer and the Caco-2 colon cancer cell lines in an MTT assay [18]. Furthermore, the MTT assay revealed a cytoprotective effect of patuletin on the human lung carcinoma GLC_4_ cell line challenged by cytotoxic sesquiterpene lactone helenalin [19]. Activation of antioxidant enzymes rather than direct free radical scavenging was suggested as a possible mechanism underlying this phenomenon. Mesaik et al. [20] demonstrated that immunomodulatory and antiarthritic potential of patuletin was associated with inhibited production of the proinflammatory cytokine TNF-*α* with no cytotoxic property. Chew et al. [21] reported that marigold-derived dietary lutein enhanced phytohemagglutinin-induced lymphocyte proliferation in mice but had no effect on interleukin-2 production or lymphocyte cytotoxicity.

Human lymphoblastoid T-cell Jurkat line is a popular model for the study of immune signaling and inflammation [22]. Jurkat cells can imitate both healthy and inflammatory T-cells in their response to oxidative metabolites, such as hydrogen peroxide [23]. Although H_2_O_2_ plays an important role in antigen-dependent lymphocyte activation [24], excessive production of H_2_O_2_ induces oxidative stress and impairs T-cell activity, leading to chronic inflammation and cell death. In the presence of oxygen in aqueous medium, hydrogen peroxide can produce additional cytotoxic reactive oxygen species (ROS), such as superoxide and peroxyl radicals [25]. To control the level of ROS, cells employ antioxidant enzymes, for example, catalase that decomposes hydrogen peroxide, and superoxide dismutase neutralizing superoxide radicals, as well as low-molecular antioxidants. The latter group includes internally produced glutathione and dietary antioxidants such as ascorbic acid and phenolic compounds. The function of the antioxidant system is maintained by additional enzymes such as glutathione reductase that restores the antioxidant capacity of oxidized glutathione. Signaling for regulation of oxidative stress and inflammatory responses involve cytokines such as anti-inflammatory and antioxidant interleukin-10 (IL-10) [26].

The oxidative stress can interfere with normal progression of cell growth and division arranged in a cell cycle. In eukaryotes, a normal cell cycle consists of four main stages: G_1_, during which a cell is metabolically active and continuously grows; S phase, during which DNA replication takes place; G_2_, during which the growth of cell continues and the cell prepares for division; and the M (mitosis) phase, during which the cell divides into two daughter cells, each with a full copy of DNA. After the M phase, the cells can enter G_1_ or G_0_, a quiescent phase. When the cell detects any defects (e.g., oxidative DNA damage) which necessitate halting the cell cycle in G_1_, cell cycle arrest occurs. Efforts to correct these problems may slow growth and induce cell death [27].

The response of Jurkat cells to H_2_O_2_ is dose-dependent. Reversible oxidative changes that could be repaired by cellular antioxidant systems occurred at a H_2_O_2_ concentration of 20 *μ*M, while the signs of apoptosis (programmed cell death) were noted at 50 *μ*M H_2_O_2_ [28]. Both apoptosis and necrosis (a nonprogrammed cell death caused by damage) were observed in the Jurkat cells exposed to 100 *μ*M H_2_O_2_ [29], whereas the necrosis prevailed at 500 *μ*M H_2_O_2_ [30].

The balance between prooxidant and antioxidant repair mechanisms determining cellular survival and function can be affected by dietary bioactive compounds possessing radical-scavenging and anti-inflammatory activity. Therefore, in the present work, we investigated the effects of anti-inflammatory* T. patula* flower extract and of its purified fractions on the behavior of the H_2_O_2_-challenged Jurkat cells.

## 2. Materials and Methods

### 2.1. Plant Material

The flowers were collected from the plants of a local Georgian landrace of* T. patula* grown at experimental plot near Tbilisi from seeds purchased from a commercial supplier. The collected flowers were air-dried in the shade at 25–30°C. The dried matter was stored in a closed glass container in a cool, dry place.

### 2.2. Extraction and Purification of Marigold Constituents

The isolation was performed by sequential solvent extraction of* T. patula* flowers. A sample of 600 g of dried pulverized plant material was extracted with 1,2-dichloroethane in a Soxhlet apparatus for 48 h until color loss. The residue after the dichloroethane extraction was reextracted with ethanol (solvent/plant matter ratio 1 : 5) for isolation of compounds of higher polarity. The solvents were evaporated under vacuum at 40°C giving dichloroethane and ethanol crude extracts. Further separation of individual compounds from the dichloroethane extract was performed by column chromatography on silica gel column with chloroform-hexane solvent system. The elution of fractions from the column was started with hexane with further increase of chloroform content in the system. The elution with 3% chloroform in hexane gave compound 1. Compound 2 was present in the fraction eluted from the column with 5% chloroform in hexane.

The ethanolic extract was separated on a silica gel column by elution with dichloroethane/methanol using thin-layer chromatography (TLC) for preliminary characterization of fractions. The elution was started with dichloroethane with subsequent stepwise increase of methanol content in the system. Elution with 2, 3, 5, 7, and 10% methanol in dichloroethane produced fractions 1, 2, 3, 4, and 5, respectively. Rechromatography of fraction 2 on a Sephadex LH-20 column with 2% methanol in chloroform with further TLC separation produced compound 2 that was also found in the dichloroethane extract. Compound 3 was obtained by rechromatography of fraction 5 on a silica gel column eluted with 8% methanol in chloroform and further purified on a polyamide column with elution with aqueous ethanol.

The TLC separation was performed using silica gel plates Merck (Germany). Separation of lipophilic compounds was performed in the solvent systems of dichloroethane-methanol (9 : 1) and chloroform-methanol (9 : 1). More polar compounds from ethanolic extracts were separated in the solvent systems of chloroform/methanol/water (26 : 14 : 3). The chromatograms were inspected under UV light of 254 and 360 nm, before and after applying staining reagents for flavonoids detection. Flavonoids were detected as yellow spots revealed after heating the plates sprayed with 1% ethanolic solution of aluminium chloride. Other compounds were detected by spraying 20% sulfuric acid solutions. After heating the sprayed plates to 100°C the compounds were revealed as spots of blue to green shades, depending on specific compounds.

### 2.3. Liquid Chromatography-Mass Spectrometry (LC-MS) Analysis

The samples were dissolved in HPLC-grade methanol and filtered through a Millex-HV Durapore (PVDF) membrane (0.22 *μ*m) before being injected into the LC-MS instrument. Mass spectral analyses were carried out using the Ultraperformance LC-Quadruple Time of Flight (UPLC-QTOF) instrument (Waters Premier QTOF, Milford MA, USA), with the UPLC column connected online to a PDA detector and then to an MS detector equipped with an electrospray ion (ESI) source (used in ESI-positive mode). Separation was performed on a 2.1 × 50 mm i.d., 1.7 *μ*m UPLC BEH C18 column (Waters Acquity).

The chromatographic and MS parameters were as follows: the mobile phase consisted of 0.1% formic acid in water (phase A) and 0.1% formic acid in acetonitrile (phase B). The linear gradient program was as follows: 100% to 95% A over 0.1 min, 95% to 5% A over 9.7 min, held at 5% A over 3.2 min, and then returned to the initial conditions (95% A) in 4.2 min. The flow rate was 0.3 mL min^−1^ and the column was kept at 35°C. Masses of the eluted compounds were detected with a QTOF Premier MS instrument. The UPLC-MS runs were carried out at the following settings: capillary voltage of 2.8 kV, cone voltage of 30 eV, and collision energy of 5 eV. Argon was used as the collision gas. The *m*/*z* range was 70 to 1,000 D. The MS system was calibrated using sodium formate and Leu-enkephalin was used as the lock mass. The MassLynx software version 4.1 (Waters) was used to control the instrument and calculate accurate masses. The compounds were identified using the molecular formulae calculated on the basis of accurate mass and isotopic pattern information, and UV/visible spectra, in comparison with authentic standards of quercetin, quercetagetin, and quercetagetin-7-glucoside (Extrasynthese, Genay, France).

### 2.4. Cell Culture and Experimental Design

The human T-cell leukemia lymphoblastoid Jurkat cells (DSMZ ACC 282) were obtained from the Deutsche Sammlung von Mikroorganismen und Zellkulturen (DSMZ, Braunschweig, Germany). The cells were grown in suspension culture at 37°C under 5% humidified CO_2_ in bioactive medium RPMI 1640 (Gibco, Grand Island, NY, USA) containing inactivated embryonic bovine serum (Sigma, St Louis MO, USA), L-glutamine (4 mM), penicillin (100 U mL^−1^), and streptomycin (100 U mL^−1^). The experiments were carried out at cell densities of 0.3 to 0.6 × 10^6^ cells mL^−1^. In order to imitate the oxidative stress conditions, H_2_O_2_ (Sigma) was added to the Jurkat culture to reach the concentrations of 25 and 50 *μ*M, corresponding to low and intermediate stress severity, respectively [28]. In the unstressed control treatment, water was added to the samples instead of H_2_O_2_. The crude marigold extracts and isolated fractions were added to the cultures at a rate of 2 mg mL^−1^, if not specified differently in the text.

### 2.5. Electron Paramagnetic Resonance (EPR) Spectroscopy

The effect of marigold extracts on the generation of free radicals in H_2_O_2_-challenged and unchallenged cells was studied using the electron paramagnetic resonance (EPR) method. EPR spectra were registered on a radiospectrometer, RE 1307 (EPSI, Chernogolovka, Russia). Peroxyl radicals were detected with spin-trap *α*-phenyl-tert-butylnitrone (PBN; Sigma) (50 mM on 0.6 × 10^6^ cells in 0.5 mL medium) at room temperature at microwave power (20 mV). Superoxide radicals were detected with a spin-trap 5,5-dimethyl-I-pyrrole-IV-oxide (DMPO) (Sigma) (50 mM on 0.6 × 10^6^ cells in 0.5 mL medium) at room temperature at microwave power (20 mV).

### 2.6. Cell Viability

Cell viability was assayed by the MTT test based on evaluating cellular dehydrogenase activity [31]. Cell suspensions (2 × 10^6^ cells mL^−1^) were incubated with H_2_O_2_ and marigold preparations as described above. After the incubation period, the cells were harvested by centrifugation at 1500 g for 5 minutes, washed, and resuspended in fresh medium. The 8 mg mL^−1^ solution of 3-(4,5-dimethylthiazol-2)-2,5-diphenyltetrazolium bromide (MTT) (Sigma) in buffer (140 mM NaCl, 5 mM HEPES, pH 7.4) was added to the cell suspension at a rate of 30 *μ*L per 100 *μ*L suspension and the mixture was incubated for 4 h at 37°C in a 5% CO_2_ atmosphere. After this incubation, the supernatant was carefully removed and the colored formazan crystals produced from the MTT were dissolved in 100 *μ*L of dimethyl sulfoxide (DMSO). The absorption values of the solutions reflecting the cellular dehydrogenase activity were measured at 570 nm. The effects of various treatments on cellular activity and viability were expressed as percentages of their absorption values related to those of nontreated cells.

### 2.7. Cell Cycle Analysis

Cell distribution into cell cycle phases was studied by flow cytometry using propidium iodide staining [32]. The method is based on the capacity of propidium iodide to intercalate with double-stranded DNA giving information about the DNA distribution between the cell cycle phases. The cells were fixed in 70% ethanol at 40°С for 12 hours. After removal of the ethanol, 100 *μ*g mL^−1^ RNAase (Sigma) was added to the cellular pellet and incubated for 30 minutes at room temperature. After suspending the cells with propidium iodide solution, they were incubated for 30 minutes at room temperature and analyzed by flow cytometry (excitation and emission at 488 and 617 nm, resp.) in accordance with the DNA content as follows: (a) haploid state-apoptotic cells; (b) diploid state-cells in G_0_/G_1_ phase; (c) transitional state between diploid and tetraploid-cells in phase S, (d) tetraploid state-cells in G_2_/M phase. The percentage of cells in each state was determined.

In addition, a percentage of apoptotic cells in the population (apoptotic ratio) was determined by flow cytometry on the basis of mitochondrial transmembrane potential (ΔΨ) measurement using a lipophilic cation test with 3,3′-dihexyloxacarbocyanine iodide (DiOC_6_) [33]. In order to determine the mitochondrial potential, 10^5^ cells were incubated with 120 *μ*L of 0.2 *μ*M DiOC_6_ solution for 15 min at 37°C. The studies were conducted using the FACSCalibur flow cytometer (Becton Dickinson, Franklin Lakes, NJ, US); excitation and emission wavelengths for DiOC_6_ were 488 and 530 nm, respectively.

The structure of the Jurkat cells was investigated under transmission electron microscope (TEM) Tesla BS 500 (Tesla, Brno, Czech Republic) after fixation with 2.5% glutaraldehyde (pH 7.4) and toluidine blue staining.

### 2.8. Antioxidant Enzymes

Jurkat cell extract was prepared by centrifugation of the cell suspensions at 500 g and then homogenizing the cellular precipitate in a lysis buffer (pH 7.9) that was comprised of 1.5 mM MgCl_2_, 10 mM KCl, 1 mM dithiothreitol, 1 *μ*g mL^−1^ leupeptin, 1 *μ*g mL^−1^ aprotinin, and 10 mM HEPES. The volume of the buffer was twice the volume of the precipitate. Lysis of the cells was performed by passing the suspension through a 27-gauge needle 10 times. The obtained homogenate was centrifuged for 20 min at 10,000 g. The supernatant was used to determine the levels of enzyme activity. Catalase (EC 1.11.1.6) activity was measured spectrophotometrically as the decomposition of H_2_O_2_ at 240 nm [23]. One unit of catalase activity was defined as the amount of enzyme decomposing 1 *μ*mol of H_2_O_2_ per minute. Superoxide dismutase (SOD; EC 1.15.1.1) was assayed using NADPH and phenazine methosulfate (PMS) reagents for the reduction of nitroblue tetrazolium salt (NBT) into blue-colored formazan measured spectrophotometrically at 560 nm [24]. One unit of SOD activity was defined as the amount of enzyme oxidizing 1 nmol NADPH per minute. Glutathione reductase (GR; EC 1.8.1.7) activity was measured spectrophotometrically as oxidation of NADPH monitored at 340 nm in the presence of oxidized glutathione. Glutathione reductase activity was expressed as nmol substrate oxidized per minute. The activity of the enzymes was expressed in terms of units per mg of protein. A total protein micro-Lowry kit (Sigma) was used to determine the protein content.

### 2.9. ORAC Assay of Nonenzymatic Radical-Scavenging Activity

Dried marigold petals were extracted by stepwise extraction with acetate buffer, acetone, and hexane and repeated partition of water-soluble and water-insoluble portions as described by Vinokur and Rodov [34]. The hydrophilic water/acetone fraction was used for measuring the oxygen radical absorbance capacity (ORAC) according to the procedure described by Gillespie et al. [35]. The assay is based on measuring the degradation of a fluorescent probe by free radicals, resulting in decline of its fluorescence intensity. The radical-scavenging efficacy of antioxidants is assessed by delay of the fluorescence decay in comparison with a standard antioxidant Trolox (6-hydroxy-2,5,7,8-tetramethylchroman-2-carboxylic acid, Sigma). Fluorescein (Sigma) 0.08 *μ*M was used as a fluorescent probe. Peroxyl radicals were generated by 150 mM of azo-initiator 2,2′-azobis(2-amidinopropane) dihydrochloride (AAPH) (Sigma) at 37°C. A SPEX fluorometer (SPEX Industries, Edison NJ, USA) was used for fluorescence measurement at excitation and emission wavelengths of 485 and 530 nm, respectively.

### 2.10. Interleukin Analysis

Jurkat cells were prestimulated by incubation with 50 *μ*g/mL phytohemagglutinin (PHA) at 37°C for 5 min and cultured for 24 h with nonstimulated Jurkat cells (40% stimulated and 60% nonstimulated cells). The level of anti-inflammatory and antioxidant cytokine IL-10 was assayed using ELISA kit (Bender Medsystems, Vienna, Austria) and the Multiscan microplate reader (LabSystem, Helsinki, Finland).

### 2.11. Statistics

The trials were performed in five replications. The statistical analysis of the obtained results, including calculation of means and standard deviations, was conducted using the IBM SPSS Statistics program. The statistical significance of the differences between the treatment results versus nontreated control was analyzed by pairwise comparison using Student's *t*-test at *P* values of ≤0.001, ≤0.01, and ≤0.05 designated as *∗∗∗*, *∗∗*, and *∗*, respectively.

## 3. Results and Discussion

### 3.1. Composition of* T. patula* Extracts

**Figure 1:**
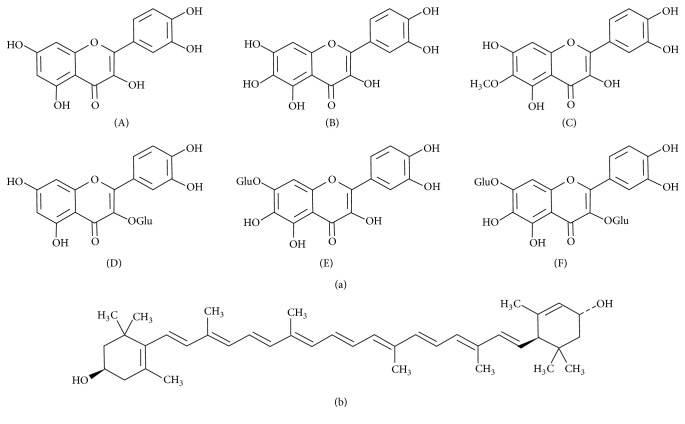
The constituents of* Tagetes patula*. (a) Flavonols: (A) quercetin; (B) quercetagetin; (C) patuletin; (D) quercetin-3-glucoside; (E) quercetagetin-7-glucoside; (F) quercetagetin-3,7-diglucoside. (b) Lutein.

Several flavonoids, all belonging to the group of flavonols (Figure 1(a), (A–F)), were identified in the marigold extracts. Compound 2 was identified as patuletin (6-methoxyquercetin) with protonated molecule mass [M+H]^+^ at *m*/*z* 333 and compound 3 as quercetagetin (6-hydroxyquercetin), [M+H]^+^ at *m*/*z* 319. Quercetagetin prevailed in fraction 5. In addition, this fraction contained quercetin, glucosylated derivatives of quercetin and quercetagetin, and diglucoside of quercetagetin with protonated molecules at *m*/*z* 303, 465, 481, and 643, respectively. Such composition of flavonoids is typical to* T. patula* [36]. Compound 1 was identified on the basis of its spectral characteristics as carotenoid lutein (Figure 1(b)) known to be the major carotenoid in marigold flowers [37]. Purified compounds lutein and patulin and the flavonoid fraction rich in quercetagetin and quercetin, as well as crude marigold extracts, were used for further trials with Jurkat cells.

### 3.2. Effects on Jurkat Cell Viability

**Figure 2:**
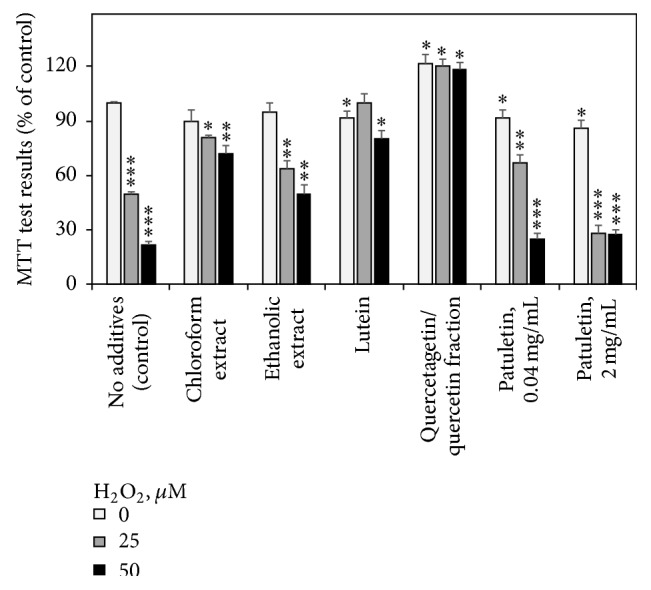
Effects of French marigold extracts and purified fractions on the results of MTT cell viability test (% of the nontreated control). Error bars represent standard deviations of five replications. Values marked with asterisks were significantly different from the nontreated control according to Student's *t*-test at *P* values of ≤0.001, ≤0.01, and ≤0.05 designated as *∗∗∗*, *∗∗*, and *∗*, respectively.

Adding marigold fractions to Jurkat cultures in the absence of exogenous H_2_O_2_ resulted just in moderate changes in MTT test results, not exceeding 20% of the nontreated cells level. Flavonoid fraction rich in quercetagetin and quercetin caused certain increase in apparent cell viability above the control level, due to the enhancement of dehydrogenase activity and/or cell proliferation (Figure 2). On the other hand, slight decline of the cell viability was observed in the presence of patuletin, in agreement with the previous observation of Woerdenbag et al. [19] who reported moderate to low cytotoxicity of this compound towards nonchallenged cells.

H_2_O_2_-induced oxidative stress reduced the viability of Jurkat cells in a dose-dependent manner. This hydrogen peroxide effect was alleviated by application of various* T. patula* fractions. The most efficient protection against H_2_O_2_ damage was rendered to the Jurkat cells by the quercetagetin/quercetin flavonoid fraction. The superiority of quercetin over other phenolic compounds in protecting Jurkat cells against H_2_O_2_-induced cell death was earlier shown by Zhang et al. [38]. Purified patuletin in the concentration of 2 mg mL^−1^ showed no cytoprotective activity and even aggravated the cytotoxic effect of H_2_O_2_. However, at low concentration of 40 *μ*g mL^−1^, patuletin did reveal certain cytoprotective activity against 25 *μ*M H_2_O_2_, increasing the cell viability from 50 to almost 70%. A similar observation was reported by Woerdenbag et al. [19] showing that moderate concentrations of flavonols protected the cells against the helenalin cytotoxicity, while in higher doses patuletin (and to a much lesser extent other flavonoids studied) turned cytotoxic by themselves. The combination of antioxidant and radical-scavenging activity of patuletin, on one hand, with its cytotoxic effect, on the other one, was described by Kashif et al. [17] in relation to its anticancer potential. Due to these findings, patuletin was applied in further trials in concentration of 40 *μ*g mL^−1^.

### 3.3. H_2_O_2_-Induced ROS Generation and Antioxidant Activity of* T. patula* Extracts

**Figure 3:**
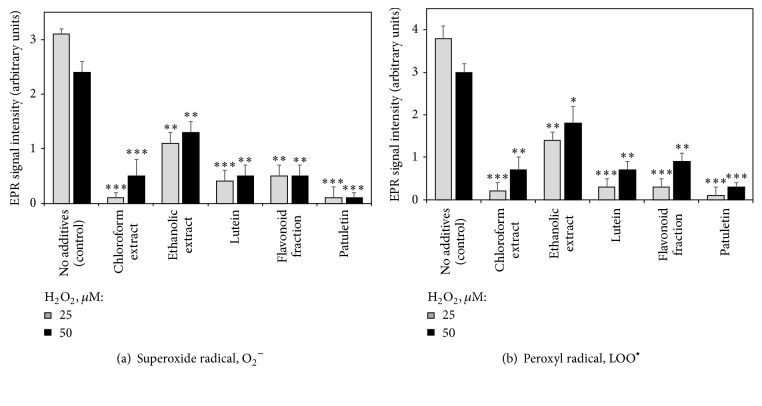
Effects of French marigold extracts and purified fractions on the generation of superoxide (a) and peroxyl (b) radicals in Jurkat cells subjected to hydrogen peroxide-induced oxidative stress (EPR signal intensity, arbitrary units). Error bars represent standard deviations of five replications. Values marked with asterisks were significantly different from the control subjected to the same H_2_O_2_ concentration, according to Student's *t*-test at *P* values of ≤0.001, ≤0.01, and ≤0.05 designated as *∗∗∗*, *∗∗*, and *∗*, respectively.

The addition of 25 or 50 *μ*M of hydrogen peroxide caused generation of superoxide and peroxyl radicals in the Jurkat culture evidenced by EPR spectroscopy (Figure 3), most probably through the mechanisms described by Petlicki and Van De Ven [25]. No radicals were detected in the absence of hydrogen peroxide (data not shown). Adding* T. patula* fractions significantly reduced the level of superoxide and peroxyl radicals in the H_2_O_2_-challenged Jurkat cultures, with purified patuletin being the most efficient radical scavenger.

**Figure 4:**
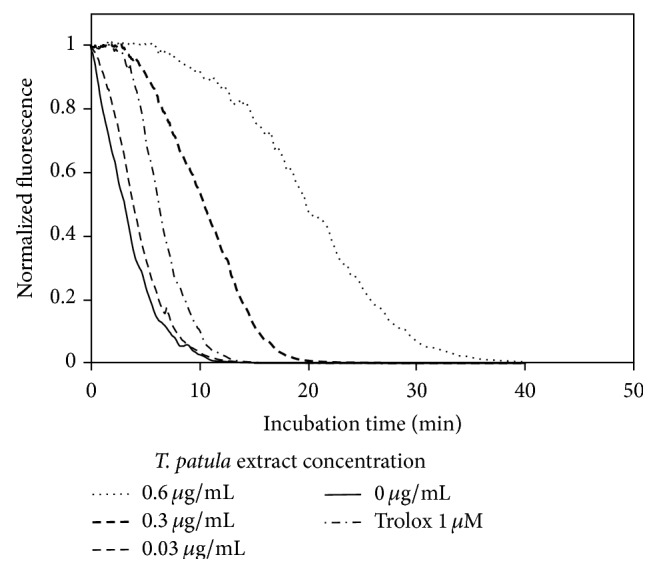
ORAC assay: representative fluorescence decay curves of fluorescein in the presence of different marigold extract concentrations and of the standard antioxidant Trolox.

The radical-scavenging capacity of* T. patula* extract was confirmed by the ORAC assay (Figure 4). Figure 4 exemplifies the protective effect of* T. patula* extract, in comparison with a standard antioxidant Trolox, against fluorescent probe degradation caused by AAPH-generated peroxyl radicals. The ORAC value of the dried marigold petals was calculated as 177.9 ± 2.8 *μ*M Trolox equivalent g^−1^, comparable with potent antioxidant spices, such as paprika, black pepper, and curry [39].

**Figure 5:**
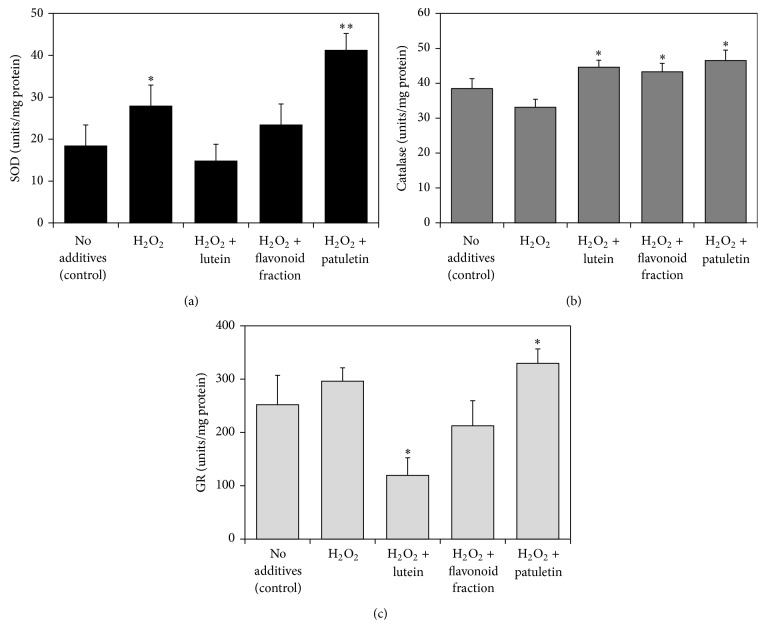
Effect of hydrogen peroxide and French marigold extract fractions on the activity of antioxidant enzymes in Jurkat T-cells. (a) Superoxide dismutase (SOD); (b) catalase; (c) glutathione reductase (GR). Error bars represent standard deviations of five replications. Values marked with asterisks were significantly different from the nontreated control in the same series, according to Student's *t*-test at *P* values of ≤0.01 and ≤0.05 designated as *∗∗* and *∗*, respectively.

Figure 5 presents the effect of marigold fractions on the activity of antioxidant enzymes superoxide dismutase (SOD), catalase, and glutathione reductase (GR) in the Jurkat cells. The activities of the three enzymes in the H_2_O_2_-challenged cells were enhanced by addition of patuletin fraction. The most significant increase was observed with superoxide dismutase whose activity more than doubled in the presence of patuletin. In addition, the activity of catalase was stimulated by lutein and quercetagetin/quercetin fractions.

Thus, the marigold extracts could eliminate ROS and alleviate the oxidative stress in H_2_O_2_-challenged Jurkat cultures through two mechanisms: (a) nonenzymatic scavenging of free radicals as revealed in ORAC assay and (b) stimulating the activity of ROS-neutralizing antioxidant enzymes such as SOD and catalase.

### 3.4. Effects on the Cell Cycle

**Figure 6:**
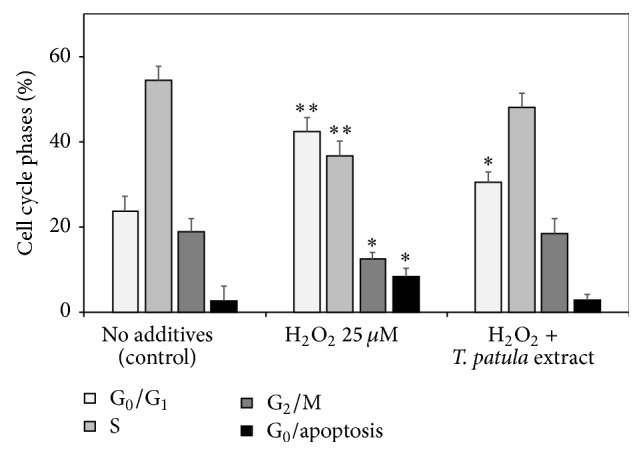
Effects of hydrogen peroxide and of the ethanolic French marigold extract on the percentage of cell cycle phase distributions of Jurkat cells (results of flow cytometry of propidium iodide-stained cell populations). Values marked with asterisks were significantly different from the respective cell cycle phase percentage in the nontreated control according to Student's *t*-test at *P* values of ≤0.01 and ≤0.05 designated as *∗∗* and *∗*, respectively.

**Figure 7:**
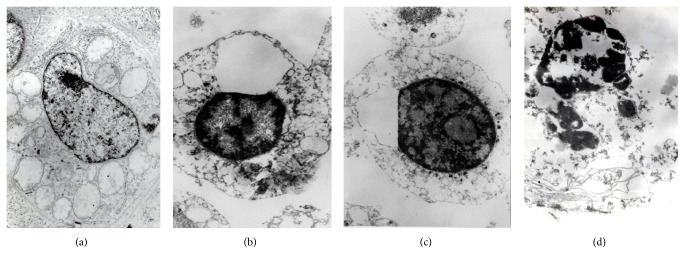
Transmission electron microscopy images of the Jurkat cells: (a) normal cell morphology; (b) and (c) early apoptosis stages in H_2_O_2_-challenged cells representing chromatin condensation and cytoplasmic vacuolization; (d) late apoptosis stage in H_2_O_2_-challenged cell representing nuclear fragmentation.

**Figure 8:**
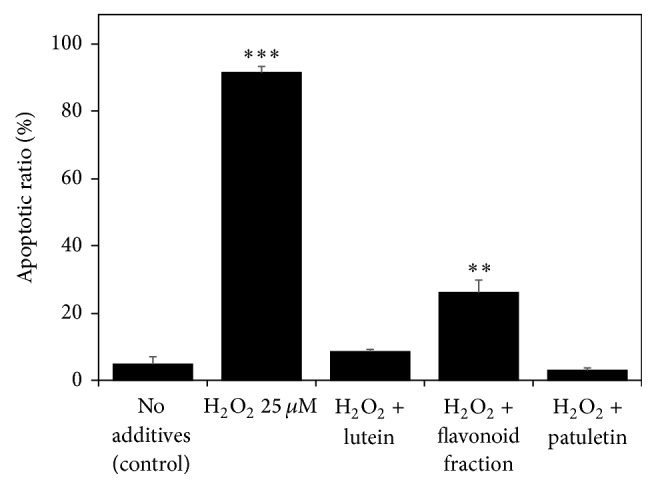
Effect of hydrogen peroxide and French marigold fractions on the percentage of apoptotic cells (apoptotic ratio) in the Jurkat cell population (results of flow cytometry based on mitochondrial transmembrane potential measurement using a DiOC_6_ staining). Error bars represent standard deviations of five replications. Values marked with asterisks were significantly different from the nontreated control according to Student's *t*-test at *P* values of ≤0.001 and ≤0.01 designated as *∗∗∗* and *∗∗*, respectively.

The H_2_O_2_-caused oxidative stress changed the cell cycle phase distribution, restricting DNA replication (phase S) and increasing the relative proportions of G_0_/G_1_ cells (the G_0_/G_1_ arrest) and apoptotic cells (Figure 6). The characteristic apoptotic changes (chromatin condensation, nuclear fragmentation, and cytoplasmic vacuolization) in typical H_2_O_2_-exposed Jurkat cells are presented in transmission electron microscope images (Figure 7). Adding the* T. patula* extract to the H_2_O_2_-challenged cells largely normalized their cell cycle (Figure 6). Similar trends were revealed by evaluating the percentage of apoptotic cells in the population (the apoptotic ratio) by flow cytometry on the basis of mitochondrial transmembrane potential measured by DiOC_6_ test. An upsurge in the apoptotic ratio was induced by hydrogen peroxide alone, while this increase was counteracted by coadministering the* T. patula* fractions containing patuletin, quercetagetin/quercetin, or lutein (Figure 8). Certain discrepancy in the proportion of apoptotic cells was evident between the two flow cytometry methods, most probably due to the different separation criteria used. Similarly, Özgen et al. [40] reported that in the T-cells, the DiOC_6_ technique gave higher estimation of apoptotic cell population as compared to the propidium iodide-based method.

### 3.5. Interleukin-10 (IL-10) Production

**Figure 9:**
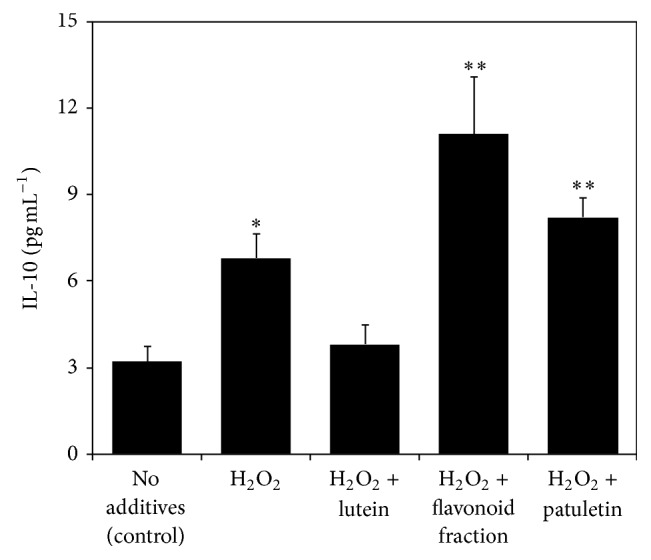
Effect of hydrogen peroxide and French marigold fractions on the level of interleukin-10 (IL-10) in the Jurkat cells. Error bars represent standard deviations of five replications. Values marked with asterisks were significantly different from the nontreated control according to Student's *t*-test at *P* values of ≤0.01 and ≤0.05 designated as *∗∗* and *∗*, respectively.

Anti-inflammatory and antioxidant interleukin-10 (IL-10) increased in the Jurkat cells subjected to H_2_O_2_ challenge, most probably as a part of a defense mechanism against oxidative stress. Similar response was observed in cultured human keratinocytes exposed to ultraviolet irradiation [41]. Flavonoid fractions of* T. patula* further enhanced the IL-10 level in the H_2_O_2_-challenged Jurkat cells (Figure 9), similar to our previous work with* Satureja hortensis* [42]. The IL-10 is known to inhibit the apoptotic death of T-cells, presumably through upregulation of Bcl-2 [26]. This mechanism might underlay the alleviation of apoptosis observed in our study. On a whole organism level, the diet rich in phenolic antioxidants enhanced the IL-10 production in the animals enduring proinflammatory conditions, resulting in a decrease in proinflammatory factors, inhibited lipid peroxidation, increased HDL levels, and alleviated inflammation [43].

## 4. Conclusions

The present research has demonstrated for the first time that both flavonoid and carotenoid constituents of French marigold (*Tagetes patula* L.) extract can protect Jurkat cells from hydrogen peroxide caused oxidative stress. Both direct radical-scavenging effects and stimulation of the cellular antioxidant enzymes and anti-inflammatory factors such as IL-10 can be involved in these protective mechanisms. The findings are in line with the antioxidant and anti-inflammatory properties of marigold preparations used in folk medicine and confirmed in animal and human studies. At the same time, it was found that some* T. patula* flavonoids, primarily patuletin, can exert cytotoxic effect on Jurkat cells associated with its anticancer potential. The shift between cytoprotective and cytotoxic activity depends on concentration and chemical nature of the compound.
